# A novel preparative method for nanoparticle albumin-bound paclitaxel with high drug loading and its evaluation both *in vitro* and *in vivo*

**DOI:** 10.1371/journal.pone.0250670

**Published:** 2021-04-28

**Authors:** Yue Gao, Jingxue Nai, Zhenbo Yang, Jinbang Zhang, Siyu Ma, Yumei Zhao, Hui Li, Jiaxin Li, Yang Yang, Meiyan Yang, Yuli Wang, Wei Gong, Fanglin Yu, Chunsheng Gao, Zhiping Li, Xingguo Mei

**Affiliations:** 1 Department of Pharmaceutics, State key Laboratory of Toxicology and Medical Countermeasure, Beijing Institute of Pharmacology and Toxicology, Beijing, China; 2 Institute of Materia Medica, Chinese Academy of Medical Sciences & Peking Union Medical College, Beijing, China; 3 Wuhan University School of Pharmaceutical Sciences, Wuhan, China; 4 Pharmaceutical College of Henan University, Kaifeng, China; St. John’s University, UNITED STATES

## Abstract

We developed a novel preparative method for nanoparticle albumin-bound (nab) paclitaxel with high drug loading, which was based on improved paclitaxel solubility in polyethylene glycol (PEG) and self-assembly of paclitaxel in PEG with albumin powders into nanoparticles. That is, paclitaxel and PEG were firstly dissolved in ethanol, which was subsequently evaporated under vacuum. The obtained liquid was then mixed with human serum albumin powders. Thereafter, the mixtures were added into phosphate-buffered saline and nab paclitaxel suspensions emerged after ultrasound. Nab paclitaxel was finally acquired after dialysis and freeze drying. The drug loading of about 15% (W/V) were realized in self-made nab paclitaxel, which was increased by approximately 50% compared to 10% (W/V) in Abraxane. Now this new preparative method has been authorized to obtain patent from China and Japan. The similar characteristics of self-made nab paclitaxel compared to Abraxane were observed in morphology, encapsulation efficiency, *in vitro* release, X-ray diffraction analysis, differential scanning calorimetry analysis, and circular dichroism spectra analysis. Consistent concentration-time curves in rats, biodistributions in mice, anti-tumor activities in mice, and histological transmutation in mice were also found between Abraxane and self-made nanoparticles. In a word, our novel preparative method for nab paclitaxel can significantly improve drug loading, obviously decrease product cost, and is considered to have potent practical value.

## Introduction

Paclitaxel, a major anticancer drug isolated from the bark of Taxus brevifolia, exhibits antineoplastic activity against a wide variety of malignancies and has been successfully used in the treatment of a wide variety of cancers, such as breast, lung, and advanced ovarian cancers [1–3]. However, the poor aqueous solubility of paclitaxel limits its application since hydrophobic drugs with low water solubilities are concomitantly associated with poor pharmacokinetic profiles, low therapeutic efficacies, and adverse effects [4]. To improve the solubility of paclitaxel, a mixture of Cremophor EL (polyoxyethylated castor oil)/ethanol is used in formulations, among which the Taxol formulation is widely used in the clinic [5]. The amount of Cremophor EL in commercial formulations of paclitaxel is significantly higher than that given with any other marketed drugs [2]. Regretfully, Cremophor EL is biologically and pharmacologically active [5] and the Taxol formulation of paclitaxel, with particularly high Cremophor EL concentrations, induces severe allergic, hypersensitivity, and anaphylactic reactions, as well as potential neuropathy [1, 2, 5]. To minimize the incidence and severity of these reactions, premedication with histamine-1 and -2 blockers, as well as glucocorticoids (usually dexamethasone), has become standard practice [6]. The cumulative side effects of dexamethasone used as a premedication may add to treatment-related morbidity and, in some instances, result in early discontinuation of therapy [7]. An additional problem arising from Cremophor /ethanol solvents is the leaching of plasticizers from PVC bags and infusion sets in routine clinical use [1, 7, 8].

Because of the inherent problems associated with Cremophor EL, some new drug delivery systems for paclitaxel are being investigated to improve its aqueous solubility and to reduce side effects. These delivery systems include emulsions [9], liposomes [10], water-soluble prodrugs [11], nanoparticles [12], cyclodextrin complexes [13], polymeric micelles [14–16], and nanoparticle-colloidal suspensions [17]. Among these, the most successful formulation is Abraxane, a novel human serum albumin (HSA)-bound nanoparticle paclitaxel formulation which has been approved by FDA [17]. By using Abraxane in the absence of Cremophor EL, the risk of hypersensitivity reactions is decreased significantly, and steroidal and antihistamine premedication can thus be cancelled and the danger of leaching plasticizers from infusion bags or tubing can be avoided [5, 7, 18]. The volume and time required for administration is also reduced because Abraxane can be reconstituted in normal saline at higher concentrations of 2-10mg/mL compared with 0.3–1.2mg/mL for Taxol [7]. Moreover, the employed drug concentration for ameliorating tumors is increased through enhanced permeability and retention [19] in combination with receptor-activated transcytosis [20] when Abraxane is used, and the maximum tolerated doses are enhanced compared with those of Taxol [7]. Therefore, Abraxane represents an improvement in paclitaxel formulations and serves as a landmark not just for albumin-based drug-delivery technology, but also nanomedicines in general [21].

Abraxane is produced by patented nanoparticle albumin-bound (nab) technology, which is based on an emulsion-evaporation cross-linking method [22]. In brief, the oil phase (chloroform and ethanol) containing paclitaxel is first added to the aqueous phase (1% HSA solution pre-saturated with 1% chloroform). Then, the emulsion is formed after the mixture is successively subjected to low-shear forces at a low rotating speed, homogenized at a high pressure and recycled through the homogenizer, and the albumin nanosuspensions are finally yielded after the removal of solvent and sterilization by filtering [22]. The low drug loading (approximately 10%) of nanoparticles prepared with nab technology [5] may be ascribed to corresponding drug solubility in organic solvents and limited volumes of organic solvents in the emulsion system. Moreover, the removal of organic solvents is putatively difficult since organic solvents are packaged in the inner phase of the emulsion and a high cost is required to obtain satisfactory organic solvent residues. Hence, improvements in preparative technologies for albumin nanoparticles are still needed.

In addition to nab technology, desolvation (coacervation) and emulsification followed by fixation via chemicals (usually glutaraldehyde) or thermal treatment are also classic methods for albumin-based nanoparticle formation [23]. In spite of these current loading trends, low entrapment efficiency and introduced toxicity are also predominant issues in solvent media preparation for these HSA nanoparticles [23].

**Fig 1:**
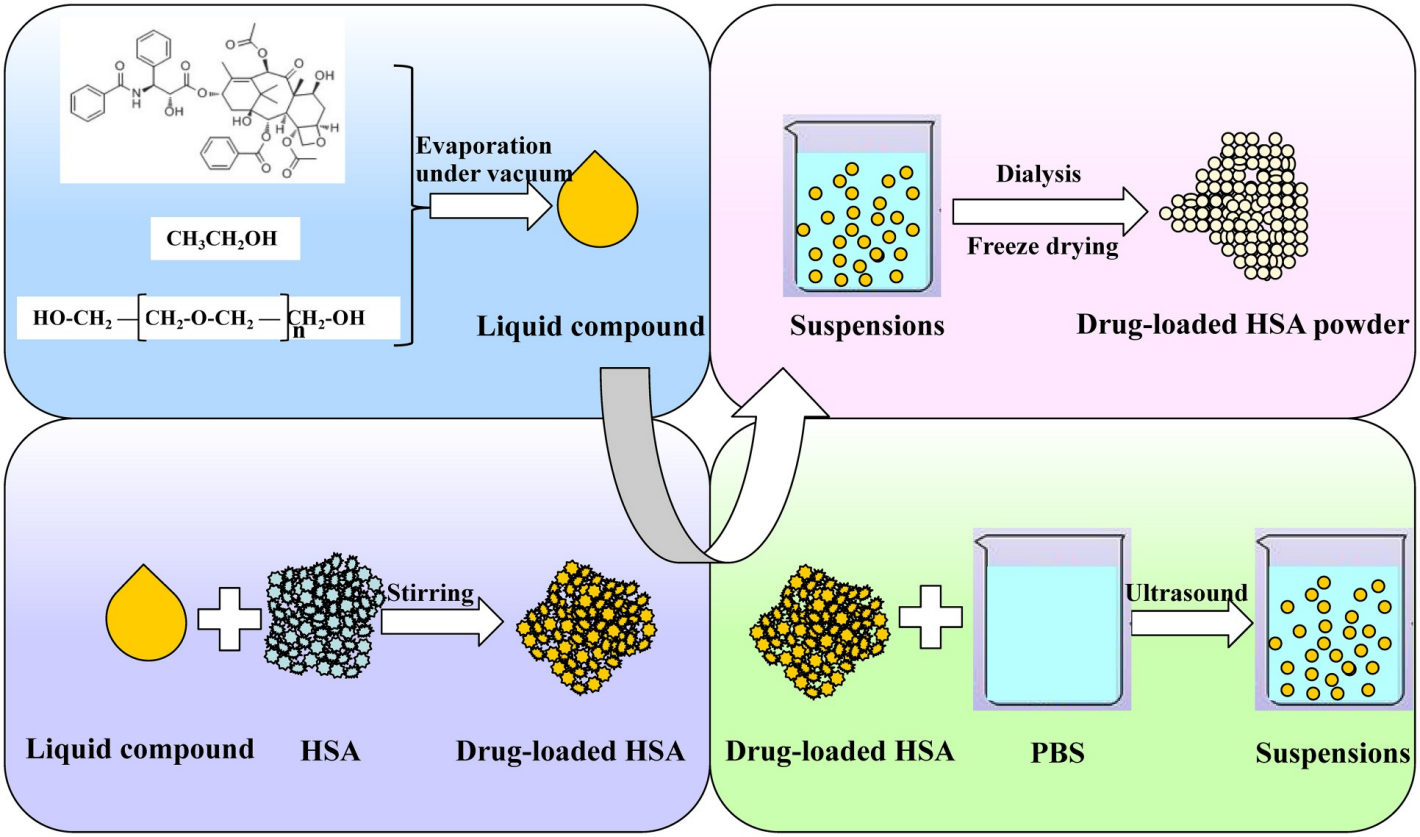
Schematic of nab paclitaxel preparation via a novel method. First, we dissolved paclitaxel and PEG in ethanol via ultrasound, and ethanol was subsequently evaporated under vacuum to obtain the liquid compound of paclitaxel and PEG. Then, paclitaxel was loaded in HSA powders by adding the liquid compound dropwise to HSA powder while stirring. Thereafter, the mixtures added to PBS under ultrasound, after which nab paclitaxel suspensions emerged. PEG and the residual ethanol in suspensions were removed by dialysis, and nab paclitaxel was finally obtained after freeze drying.

To overcome these problems, our laboratory has explored a novel method. This method is based on two pivotal findings. The first one is that the solubility of paclitaxel in polyethylene glycol (PEG) can be significantly improved when ethanol is added first to dissolve paclitaxel and PEG, after which the ethanol is removed. The other is that mixtures of paclitaxel in PEG and albumin powders can self-assemble into particles once they come into contact with water. We acquire a novel preparative method for paclitaxel albumin nanoparticles according to the above two findings and a flow chart of this new preparative method is shown in Fig 1. First, we dissolved paclitaxel and PEG in ethanol via ultrasound, and ethanol was subsequently evaporated under vacuum to obtain the liquid compound of paclitaxel and PEG. Then, paclitaxel was loaded in HSA powders by adding the liquid compound dropwise to HSA powder while stirring. Thereafter, the mixtures were added to phosphate-buffered saline (PBS) under ultrasound, after which nab paclitaxel suspensions emerged. PEG and the residual ethanol in suspensions were removed by dialysis and nab paclitaxel was finally obtained after freeze drying.

To certify the feasibility of this novel preparative technology, nab paclitaxel obtained with this new method was evaluated and compared with Abraxane both *in vitro* and *in vivo*. Collectively, our novel preparative method was easy to manipulate, and removal of ethanol (which was the only organic solvent used throughout the entire procedure) was not difficult since there were two steps available for its removal. Moreover, our findings indicate that our novel preparative method exhibited considerable improvements in drug loading over existing methods. This new preparative method has been applied for patent in China and Patent Coorperation System, and now has been authorized to obtain the invention patent from China National Intellectual Property Administration and Japan Patent Office [24, 25].

## Materials and methods

### Materials

Human serum albumin was bought from the Baxter AG Co. (Austria). PEG 400 and ethanol were obtained from the Sinopharm Chemical Reagent Co., Ltd. (Beijing, China). Paclitaxel was purchased from Hui Ang Pharmaceutical Limited Company (Guilin, China). Abraxane was produced by Abraxis BioScience, LLC (Los Angeles, USA). Taxol was produced by Corden Pharma Latina S.P.A. (Sermoneta, Italy). All other reagents were of analytical grade.

### Animals

Male Wistar rats (weighing 180-220g) and male nude mice (weighing 18-20g) were purchased from Vital River Laboratories (Beijing, China). All animals were housed in ventilated cages with free access to food and water under standardized conditions. Rats and mice were all acclimatized to laboratory conditions for 7d before the start of experiments. Additionally, a dim red light was used during the dark phase of the provided light/dark cycle. All surgeries were performed under sodium pentobarbital anesthesia and all efforts were made to relieve suffering. All animals were handled according to the code of ethics defined by the Animal Care and Use Ethics Committee of the Academy of Military Medical Sciences (Approval Number: IACUC-2016-31).

### Preparation of nab paclitaxel via a novel method

We fulfilled the preparation of nab paclitaxel by the novel method through the following three steps: (1) preparation of PEG-paclitaxel liquid compound; (2) mixing of liquid compound with albumin; and (3) dispersing of nab paclitaxel. First, paclitaxel and liquid PEG were briefly dissolved in ethanol under ultrasound, and ethanol was subsequently evaporated under rotary evaporation. The obtained liquid compounds were then mixed with HSA powders and dispersed in aqueous solution under sonication. The self-made nanoparticles were finally acquired after dialysis and lyophilization.

To further optimize our formulation and preparative technology, we investigated the effects of drug loading, ultrasonic condition, dispersing solvent, ultrasonic power, and reconstitution on nanoparticle characteristics.

### Characterization of self-made nab paclitaxel

#### Morphology, particle size, and zeta potential analysis

To characterize the nab paclitaxel made with our novel method and to determine the feasibility of this new method, we determined the morphology, particle size and zeta potential of both self-made nanoparticles and Abraxane. The morphologies of self-made nanoparticles and Abraxane were characterized by transmission electron microscopy (TEM, HITACHI, H-7650, Japan) and atomic force microscopy (AFM, NanoWizarc, JPK Ltd., Germany). For TEM characterization, the suspensions of nanoparticles in water were each dropped onto a copper grid to form a dry film at room temperature. Then, the samples were negatively stained with 2% phosphotungstic acid, air-dried at room temperature, and observed using TEM. For AFM observations, the nanoparticles were dispersed into water, spread onto a mica sheet and dried at room temperature before detection. The particle sizes of self-made nanoparticles and Abraxane were determined by photo-correlation spectroscopy (Nanophox, Sympatec GmbH, Germany) at 25°C. Measurements were performed in triplicate, and the results were reported as the mean value of 50% particle distribution. Zeta potentials of these nanoparticles were conducted by using a Malvern Nano-ZS90 (Malvern Instruments, UK) and the results are all reported as the means ± standard deviations (SD) [25].

#### Determination of residual ethanol

Residue ethanol was detected since it was used in the preparation of self-made nanoparticles. To acquire the residue level of ethanol in these nanoparticles, approximately 50mg of self-made nanoparticles was first added into 2mL of ultrapure water, and the suspensions were sonicated for 8min via rigorous sealing to ensure complete ethanol dissolution. Then, 2mL of 4% (W/V) salicylsulfonic acid was added into the above suspensions under vortex to further precipitate the nanoparticles containing albumin. The mixtures were thereafter centrifuged at 800g and 1mL of the obtained clear supernatant was placed into a 20mL headspace vial and was sealed immediately for analysis. A blank solution was obtained by mixing ultrapure water and 4% (W/V) salicylsulfonic acid at a 1:1 ratio. Working standard solutions were prepared from the standard solution containing nominal concentrations of 50g/mL of ethanol in the same way as that for the blank solution [26, 27].

All samples were detected by the gas chromatography (GC) method. An Agilent 6890N gas chromatograph equipped with an FID system and an Agilent headspace G1888 autosampler equipped with a 1.0mL sample loop, was used. The separation was performed on a Rtx-1301 capillary column (30m × 0.53mm, 3μm) at 90°C. Nitrogen was used as the carrier gas at a flow rate of 2.5mL/min. The inlet temperature was 220°C with a split ratio of 2:1 to reduce any volatile compounds of preparations into the GC system. The FID temperature was 250°C, and the temperatures of the sample loop and the transfer line were 90°C and 95°C, respectively. The equilibration was performed at 85°C for 30min. Method validation was performed and the limit of detection and limit of quantitation were 0.25μg/mL and 0.55μg/mL (equivalent to 50μg and 110μg per g of nanoparticles) respectively, which were far lower than the residue limit of ethanol.

#### Drug loading and encapsulation efficiency

The loading and encapsulation efficiency of paclitaxel in self-made nanoparticles and Abraxane were obtained by determining the amounts of free paclitaxel and total paclitaxel. To obtain the free paclitaxel in these particles, approximately 25mg of paclitaxel nanoparticles was firstly suspended in 10mL of PBS. Then, 0.5mL of the suspensions was drawn out and placed into the upper chamber of a centrifuge tube matched with an ultrafilter (Sartorius Vivaspin 500μL, 30k MWCO PES, Germany). Finally, the ultrafiltrate containing free drug was acquired after centrifugation for 30min at 12,000rpm. To determine the total drug in paclitaxel nanoparticles, approximately 10mg of self-made nanoparticles or Abraxane was added into 10mL of cold acetonitrile, followed by vortex for 5min and centrifugation at 12,000rpm for 5min. Thereafter, the supernatant was used for further analysis [28].

The amount of free paclitaxel in filtrate and the total amount of paclitaxel encapsulated and unencapsulated in nanoparticles were both detected by high-performance liquid chromatography (HPLC). The separation was performed on a reverse-phase silica column (Agela VenusiL MP-C18, 4.6×250mm, 5μm). The mobile phase consisted of acetonitrile and water (47.5: 52.5, V/V) and was pumped at a flow rate of 1.0mL/min. The detection wavelength was 227nm, and the injection volume was 20μL [29].

The encapsulation efficiency and drug loading were obtained according to Eqs (1) and (2), respectively, where W_total drug_ indicates the amount of paclitaxel in the whole nanoparticle suspension, W_free drug_ indicates the amount of paclitaxel unencapsulated in the nanoparticles, and W_nanoparticles weighed_ indicates the amount of paclitaxel nanoparticles used for determination.

$$\text{Encapsulation}\;\text{efficiency}(\%)=[{\text{W}}_{\text{total}\;\text{drug}}-{\text{W}}_{\text{free}\;\text{drug}}]/{\text{W}}_{\text{total}\;\text{drug}}\times 100\%$$ (1)

$$\text{Drug}\;\text{loading}(\%)=[{\text{W}}_{\text{total}\;\text{drug}}-{\text{W}}_{\text{free}\;\text{drug}}]/{\text{W}}_{\text{nanoparticles}\;\text{weighed}}\times 100\%$$ (2)

#### *In vitro* drug release

The *in vitro* drug release of nab paclitaxel was carried out in PBS containing 0.05% (W/V) Tween 20 and 0.05% (W/V) Tween 80 via a dialysis method. In brief, approximately 5mg of self-made nanoparticles or Abraxane was first suspended in 10mL of release media. Then, 3mL of the total was drawn out and put into dialysis bags (MWCO 8000–14000 Da, Beijing Henghui Co., Ltd., China), and these samples were finally immersed in 50mL of release medium under stirring at 37°C. The release medium was completely replaced with 50mL of fresh release media at predetermined times. The concentrations of paclitaxel in the release media were determined by HPLC [3, 23, 30]. For comparison, the release of paclitaxel from Taxol was also performed under the same conditions. All of the tests were performed in triplicate.

#### X-ray diffraction (XRD) analysis

To evaluate the dispersion state of paclitaxel in nanoparticles, XRD analysis was performed for paclitaxel powder, HSA powder, the mixture of paclitaxel powder and HSA powder, self-made nanoparticles and Abraxane via an X-ray Bruker D8 Advance instrument (Germany). The samples were scanned from 2θ values of 3–80° with a step size of 0.015° and a count time of 0.2s per step. All samples were rotated at 15rpm during the analysis. The X-ray source was Cu-Ka radiation (l = 1.5418 Å) generated at 40mA and 40kV [23].

#### Differential scanning calorimetry (DSC) analysis

DSC analysis was performed using a DSC Q2000 (TA Co., USA) to evaluate the phase transitions of paclitaxel powder, HSA powder, the mixture of paclitaxel powder and HSA powder, self-made nanoparticles and Abraxane. The curves of the samples were recorded at a heating rate of 10°C/min from 40°C to approximately 250°C. Approximately 5mg of each sample was placed in a standard aluminum pan for analysis [23].

#### Circular dichroism spectra (CD) analysis

CD was performed to analyze the changes that occurred in the secondary structure of HSA in different formulations. CD analysis was carried out on a Chirascan-Plus CD Spectrometer with a 0.1-cm quartz cell. The concentrations of HSA in free HSA solution, Abraxane suspension, and self-made nanoparticles suspension were all 0.8mg/mL in the present study. The measurements were taken in the UV region with a wavelength range of 180-260nm and a band width of 5nm, and each reported spectrum was the average of three scans [31, 32]. Secondary-structure calculations were performed by Jasco software [33].

#### Colloidal stability

Turbiscan Lab Expert was used to evaluate the colloidal stabilities of resultant nanoparticles, self-made nanoparticles, Abraxane, and their 50-fold dilutions with physiological saline or minimum essential medium (MEM) containing 10% fetal bovine serum (FBS). Samples were placed into cylindrical glass tubes and were then evaluated by Turbiscan Lab Expert at predesigned time points. The stability was evaluated at 37°C with delta transmission and delta backscattering as indexes [28].

### Pharmacokinetic study in rats

Taxol, self-made nanoparticles and Abraxane were first diluted or suspended with physiological saline to obtain samples containing 1mg/mL of paclitaxel. Then, 15 rats were randomly divided into three groups (5 rats per group) and rats in each group received Taxol, self-made nanoparticles, and Abraxane via tail vein injection at a dose of 7mg/kg. Thereafter, blood samples (200μL) were collected in pre-heparinized tubes from the retroorbital plexus of rats at 0, 5, 10, 15, and 30min, as well at 1, 2, 4, 6, 8, 12, 24, 36, 48, 72, and 84h. At each time point, plasma was separated immediately by centrifuging the blood samples at 3,000rpm for 10min, after which the samples were stored at -80°C prior to analysis [28, 34].

A 10-μL aliquot of docetaxel solution (1μg/mL) was added into 100μL of plasma as an internal standard. Then, 400μL of a mixture of acetonitrile and methyl-tertiary butyl ether (20:80, V/V) was added into the above solution and shaken for 3min. Subsequently, the mixtures were centrifuged at 13,000rpm for 10min. The organic layer was collected and dried under high vacuum for 30min. The residue was then reconstituted with 100μL of the mobile phase (0.1% formic acid and methanol, 30:70, V/V). Finally, the solution was centrifuged for 5min at 15,000rpm, after which the supernatant was used for subsequent analysis [35].

Analysis was performed via an Agilent 6410 Triple Quad LC/MS system. Separation was achieved on an Agela Venusil XBP C18 (2.1×50mm, 3μm). The sample tray was held at 25°C. The mobile phase was a mixture of 0.1% formic acid and methanol (30:70, V/V). The flow rate was 0.3mL/min. The sample injection volume was 10μL. The collision gas was argon at a pressure of 8μbar. Nitrogen was used as both the drying gas and nebulizing gas. The following detection parameters were used: 540L/h desolvation gas flow, 4kV capillary voltage, 35V cone voltage, 200°C source temperature, and 150V collision energy. Detection of ions was performed in positive ionization mode with the following transitions in multiple reaction monitoring mode (MRM): m/z 854.2→569.1 for paclitaxel and m/z 808.2→527.1 for docetaxel [35].

The terminal elimination rate constant (K_e_) was estimated via least-squares regression using values in the terminal log-linear region of plasma concentration-time curves. The terminal elimination half-life (T_1/2_) was calculated as 0.693/K_e_. The area under the curve from time zero to the last sampling time (AUC_0−t_) was determined according to the linear trapezoidal rule. The area under the curve from time zero to infinity (AUC_0−∞_) was calculated as AUC_0−t_ +C_t_/K_e_, where C_t_ is the last detectable plasma concentration and t is the time at which this concentration occurred. The relative bioavailability (F_rel_) was calculated as (AUC_T_/AUC_R_) × 100%, where AUC_T_ denotes the area under the curve from time zero to the last sampling time of tested preparations and AUC_R_ represents the area under the curve from time zero to the last sampling time of Taxol. All data were expressed as means ± SD [36, 37].

### Biodistribution study in mice

Our biodistribution study was performed in H22 tumor-bearing male nude mice. First, the mice were subcutaneously injected in the flank region with 0.1mL of cell suspension containing 1×10^7^ H22 cells, and the tumors were allowed to grow until their volumes were approximately 200-300mm^3^. Then, mice bearing H22 tumors were randomly divided into four groups (25 mice per group except 3 mice in control group), and mice in each group were treated with physiological saline, Taxol, self-made nanoparticles and Abraxane via tail vein injection at a paclitaxel dose of 20mg/kg. At 0.5, 2, 4, 8, 12, and 24h after injections, five mice in each group were sacrificed. Relevant organs (heart, liver, spleen, lung and kidney) and tumors for analysis were simultaneously excised and stored at -80°C until analysis was performed.

Five-fold physiological saline was added to pre-weighed organs and the mixtures were homogenized in an ice bath. Then, 100μL of the homogenate was accurately measured and mixed with 10μL of docetaxel solution (5μg/mL) as the internal standard. Then, drugs in organs were extracted with a mixture of acetonitrile and methyl-tertiary butyl ether (20:80, V/V) (as performed for plasma). The extracted solution was collected and dried under high vacuum. The residue was reconstituted with 100μL of mobile phase (0.1% formic acid and methanol, 30:70, V/V) after vortex and sonication. Finally, the solution was centrifuged for 10min at 13,000rpm and the supernatant were used for analysis. The paclitaxel concentrations in different organs were determined via HPLC-MS/MS, as described in a previous pharmacokinetic study [38].

### Anti-tumor efficacy in mice

The anti-tumor efficacy *in vivo* was evaluated in male nude mice bearing H22 tumors that were approximately 200mm^3^. The mice were randomly divided into ten groups (5 mice per group), and mice in each group were treated every 4d by tail vein injection with physiological saline (control group), Taxol, Abraxane and self-made nanoparticles at doses of 5, 10, and 15mg/kg.

The tumor volume was measured daily and calculated according to the equation (a×b^2^)/2, where “a” and “b” indicate the length and width of the tumor, respectively. The animals were also weighed and observed every day during the experimental period. After 17d, the mice were sacrificed, and the tumors were excised and weighed. The tumor inhibition rate was calculated based on the equation (1-M_t_/M) × 100%, where M represents the average weight of tumors in the control group, and M_t_ represents the average weight of tumors in each treated group. The excised tumors were fixed in formalin and sliced after paraffin embedding. The slices were stained with hematoxylin and eosin (H&E) for pathological analysis [28, 39].

### Statistical analysis

All data were expressed as the means ± SD unless specifically outlined. Student’s t-test or one-way analysis of variance (ANOVA) were performed for the statistical evaluation of data. Differences between groups were considered statistically significant when the probability (*p*) was less than 0.05.

## Results and discussion

### Preparation of nab paclitaxel and investigation of factors affecting nanoparticle characteristics

Effects of primary factors during formulation and preparation on the particle sizes of nanoparticles were investigated for our novel method since particle size is one of the most important characteristics of HSA nanoparticles. Encapsulation efficiencies were all above 99% in spite of the preparative conditions.

#### Effects of drug loading and reconstitution on particle size

**Fig 2:**
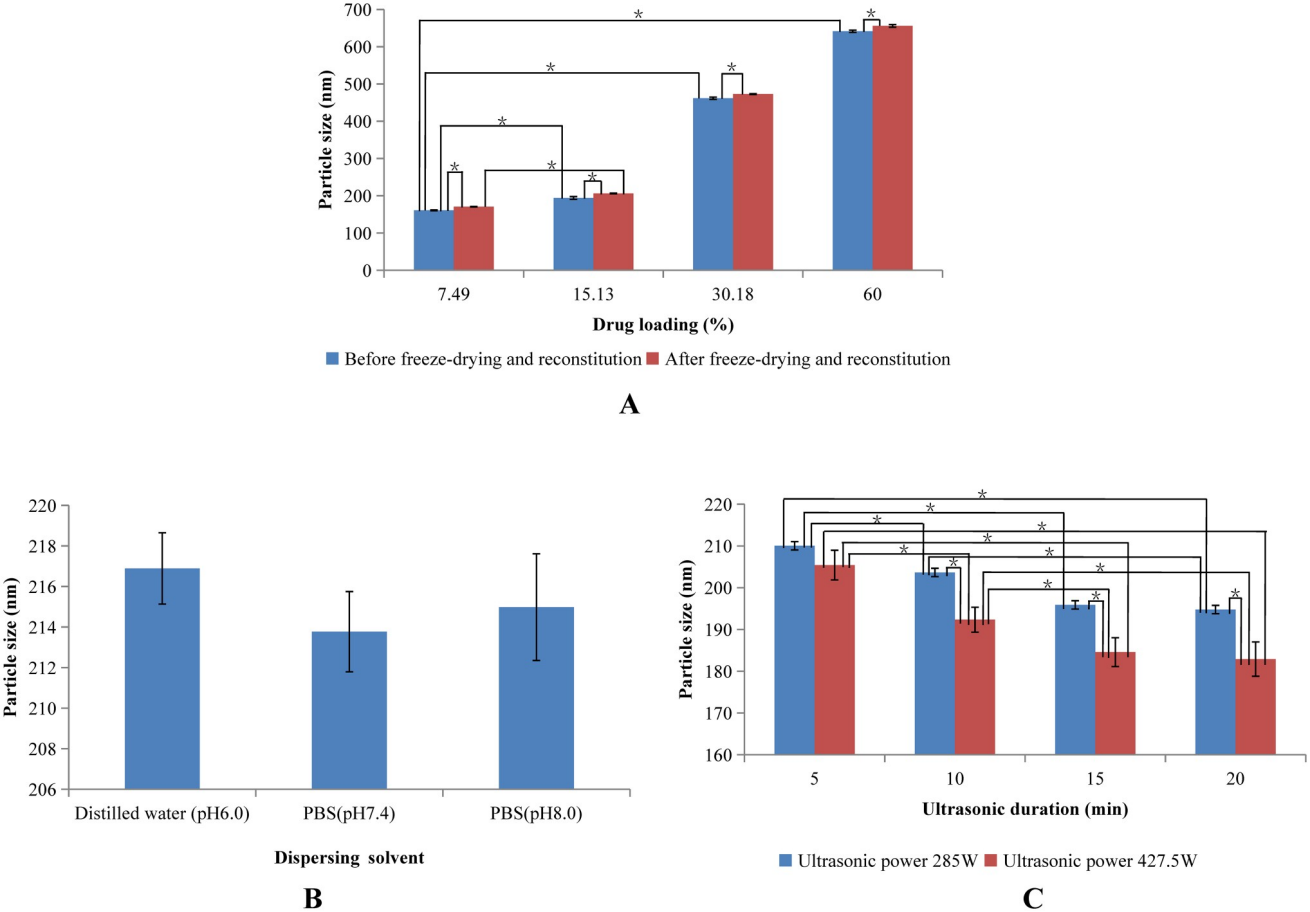
Effects of formulation and preparation on particle size of self-made nanoparticles. (A) Effects of drug loading and reconstitution on particle size. (B) Effects of dispersing solvent on particle size. (C) Effects of ultrasonic power and duration on particle size.

Since nab paclitaxel is ultimately composed of paclitaxel and HSA, the content of paclitaxel in nanoparticles is crucial. Therefore, the effect of paclitaxel content on particle size was investigated and the results are shown in Fig 2A. The particle size increased as a function of paclitaxel content. This result may have been due to the fact that the viscosity of paclitaxel-PEG complexes raises with increasing paclitaxel content, which makes it difficult to disperse mixtures of HSA and paclitaxel-PEG into aqueous solutions and consequently result in large paclitaxel-HSA particles. The highest drug loading was found to reach up to 60%. A higher drug loading with an optimal particle size is possible since our present results were obtained before optimization of our formulation and preparative method.

Self-made nanoparticles were finally produced in powder after being freeze-dried and thereby needed to be reconstituted before use. Hence, we also investigated the effect of reconstitution on particle size (Fig 2A). An improvement of approximately 10nm was found for all particles in spite of drug loading once these nanoparticles were freeze-dried and reconstituted. Therefore, freeze drying and reconstitution induced a slight increase in particle size. Nevertheless, freeze-drying and reconstitution were ultimately employed to improve the stabilities of self-made nanoparticles.

#### Effects of dispersing solvent on particle size

To inspect the effect of the dispersing solvent on particle size, 15mL of distilled water (pH 6.0), PBS (pH 7.4), and PBS (pH 8.0) were used as dispersing solvents, and the results are shown in Fig 2B. The particle sizes were approximately 215nm in spite of the types of dispersing solvents and their different pHs. Therefore, the types of dispersing solvents and their different pHs had no effect on particle size, which may have been due to each of these dispersing solvents having a pH higher than 5, the isoelectric point of HSA, and thereby had almost no effect on the charging of HSA in solvents.

To further investigate the effect of solvent volume on particle size, self-made nanoparticles were also dispersed in 10mL of PBS with a pH of 7.4. The diameter of these particles was 207.65±3.55nm, which was nearly equal to that of nanoparticles obtained from 15mL of dispersing solvent. Hence, the volume of dispersing solvent had no effect on particle size.

*Effects of ultrasonic condition on particle size*. Self-made nanoparticles were finally formed under ultrasound after mixing of paclitaxel-PEG complex and HSA in aqueous solution. The effect of ultrasonic power and ultrasonic duration on the particle size was investigated and the results are shown in Fig 2C. The size of self-made nanoparticles decreased with the increase of ultrasonic power and the same tendency was also observed for ultrasonic duration until 15min.

#### Confirmation of formulation and preparative parameters

The maximum drug loading of self-made nanoparticles was approximately 60% (W/W), and the drug loading of self-made nanoparticles with a similar particle size to that of Abraxane (approximately 145nm) was approximately 15% (W/W). Therefore, we found a high drug loading ability of self-made nanoparticles, which may be useful for reducing costs during preparation. The involved components during the whole preparing procedure were ethanol, paclitaxel, PEG, HSA, and dispersing solvent. Ethanol was added just to guarantee paclitaxel completely dissolved in liquid PEG and then was removed after paclitaxel was dissolved. PEG, a good solvent of paclitaxel, was used just as the intermediary agent of the interaction between paclitaxel and albumin, and was cleared after mixing with HSA. Therefore, PEGs with higher molecular weights such as PEG 600 could not be applied here because they always exist in solid state at room temperature. The amount of PEG was not systematically investigated since PEG was expected to be as much as possible to obtain the high drug loading while the amount of PEG was limited by the adsorption capacity of albumin.

The detailed formulation and preparative procedure were as follows. First, approximately 45mg of paclitaxel and 300μL of PEG400 were added into 10mL of dehydrated ethanol, and the mixtures were treated with sonication to dissolve paclitaxel. The liquid compound containing paclitaxel and PEG was obtained after evaporation of ethanol under negative pressure using a rotary evaporator at 65°C [23]. Second, the liquid compound was added dropwise into 240mg of HSA powders powder while stirring at room temperature to ensure complete adsorption of the liquid compound onto the HSA powder. Finally, the mixtures were added into 10mL of PBS (pH7.4) and the suspensions were then further dispersed using a probe sonicator with a power of 285W for 7.5min to form nanoparticles. The resulting nanoparticles were thereafter dialyzed with a molecular weight cutoff of 5000 in approximately 500mL of distilled water, and nab paclitaxel was finally recovered after lyophilization.

Three batches of self-made nanoparticles were prepared according to the optimized formulation and preparative parameters. It was shown that two key characteristics of the self-made nanoparticles—namely drug loading and particle size—were nearly equivalent among the three batches, and thereby the reproducibility of this new method was considered realizable.

The mechanisms of drug loading into/onto albumin nanoparticles consist of the following three types: (1) the noncovalent reversible binding of drugs with sites from HSA homologous domains or attachment through electrostatic adsorption; (2) the physical entrapment of drugs in multiple HSA molecules during the formation of nanoparticles when using methods such as emulsification and nab technology; and (3) covalent binding between HSA and drug molecules by interactions among functional groups [23]. Furthermore, drugs may be loaded into/onto albumin nanoparticles via a combination of these three mechanisms. Paclitaxel loading into/onto albumin nanoparticles with our novel preparative method may be due to noncovalent reversible binding of paclitaxel with hydrophobic sites from HSA homologous domains since the highest drug loading reached approximately 60% and there was no chemical reaction involved throughout the preparative process.

### Characterization of self-made nanoparticles

#### Morphology, particle size, zeta potential, and ethanol residue analysis

**Fig 3:**
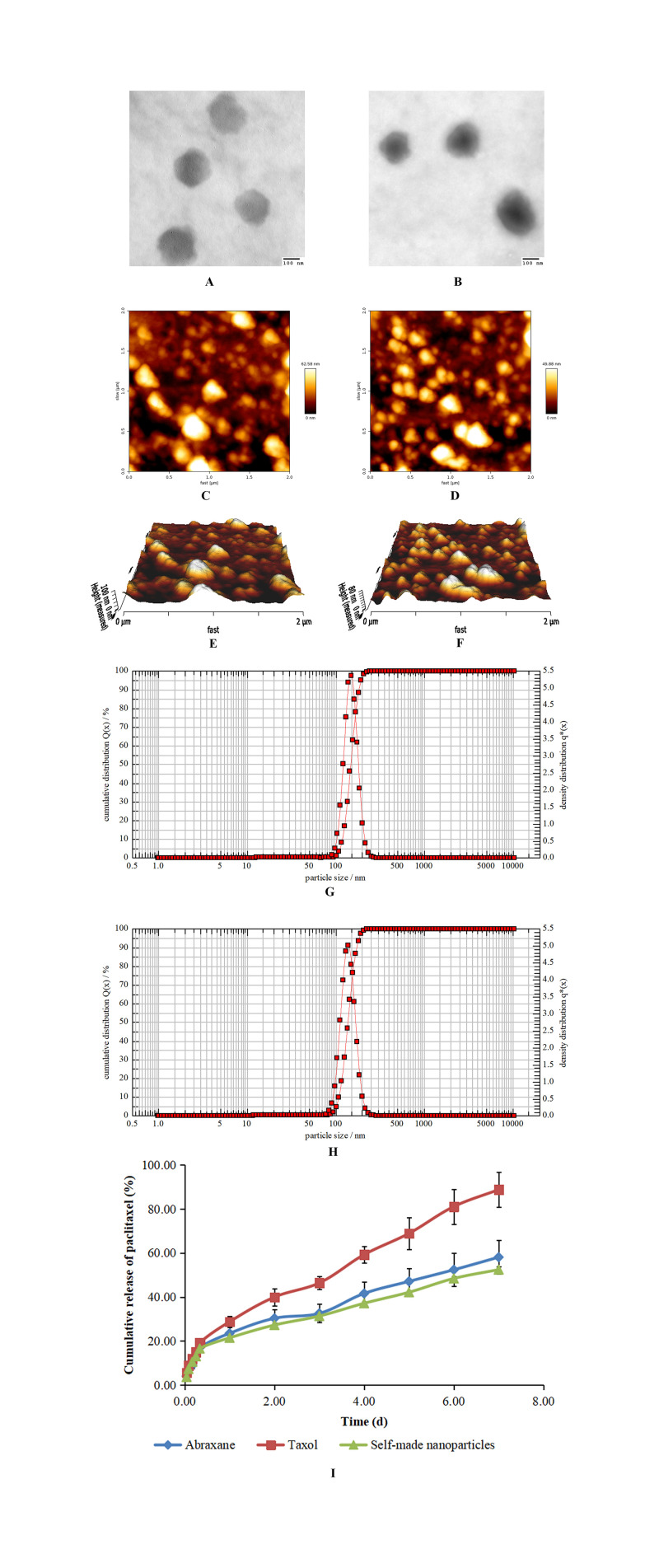
Characterization of paclitaxel nanoparticles. (A) TEM image of Abraxane. (B) TEM image of self-made nanoparticles. (C) AFM plane surface image of Abraxane. (D) AFM plane surface image of self-made nanoparticles. (E) AFM 3D image of Abraxane. (F) AFM 3D image of self-made nanoparticles. (G) Particle size distribution of Abraxane. (H) Particle size distribution of self-made nanoparticles. (I) Cumulative release profiles of paclitaxel from Taxol, Abraxane and self-made nanoparticles in PBS (pH7.4) containing 0.05% Tween 20 and 0.05% Tween 80 at 37°C.

The resulting nanoparticles were observed by both TEM and AFM, and the results are shown in Fig 3. Both self-made nanoparticles and Abraxane were round and there were no morphological differences between these two kinds of nanoparticles. The particle sizes of nanoparticles were measured with photo correlation spectroscopy and the results are shown in Fig 3G and 3H. The diameters of Abraxane and self-made nanoparticles were 146.2±1.1nm and 141.4±1.6nm, respectively, and results obtained from TEM and AFM were consistent with one another. The diameter of Abraxane measured in the present study was slightly larger than that reported previously, which may be related to different detection methods or different detection instruments. The zeta potentials of Abraxane and self-made nanoparticles were -26.4±1.2mV and -32.9±0.9mV, respectively. The negative charges of these two kinds of nanoparticles in PBS (pH7.4) were related to the isoelectric point of HSA, and this negative charge helps to maintain stability of nanoparticles *in vivo*. Ethanol residue was not detected in self-made nanoparticles, which was perhaps related to the two-step removal of ethanol during the preparative procedure.

#### Drug loading, encapsulation efficiency, and *in vitro* release

The drug loadings of paclitaxel in Abraxane and self-made nanoparticles were 9.72±0.09% and 15.29±0.10%, respectively, and the encapsulation efficiencies were 99.83±0.03% and 99.86±0.02%, respectively. It was obvious that the drug loading of paclitaxel in self-made nanoparticles was significantly improved and there was a 50% increase of drug loading in self-made nanoparticles than that in Abraxane while the encapsulation efficiencies of paclitaxel in both kinds of nanoparticles were nearly identical. These mean that the amount of HSA could be greatly reduced through this new method to obtain nanoparticles with the same paclitaxel content and thus the product cost of nab paclitaxel could be significantly decreased.

The release of paclitaxel from Taxol, Abraxane, and self-made nanoparticles at 37°C is depicted in Fig 3I. The initial bursts of paclitaxel from Taxol, Abraxane, and self-made nanoparticles at 1h were 5.65%, 4.00%, and 3.64%, respectively. In the following 7d, paclitaxel was continuously released in a sustained manner from all formulations; the cumulative release of paclitaxel reached 90% for Taxol, while the cumulative releases of paclitaxel from Abraxane and self-made nanoparticles were both lower than 60%. The incomplete release of paclitaxel from different formulations was also reported [28, 38, 40], which may have been related to the encapsulation and entrapment of paclitaxel in nanoparticles and the high affinity between paclitaxel and albumin. The release rates of paclitaxel from Abraxane and self-made nanoparticles were significantly slower than that from Taxol, while there was no difference between the release behavior of Abraxane and self-made nanoparticles since the similarity factor (*f*_2_) of these two release curves was above 50 (which is suggested by the FDA to estimate the similarity of two release profiles) [41].

The faster release of paclitaxel from Taxol compared with those from Abraxane and self-made nanoparticles was perhaps related to its molecular state since considerable quantities of ethanol and Cremophor EL were added into the formulation to dissolve paclitaxel [1, 2, 5]. The slower release rates of paclitaxel from Abraxane and self-made nanoparticles may also have been due to the slow release of paclitaxel from the complex of paclitaxel and HSA because of their high affinity. In addition, the low initial release burst and the sustained release of all formulations were also thought to be related to the dialysis release method, at least to some extent. In this release method, the dialysis membrane itself may function as a diffusion barrier (especially for poorly water-soluble drugs), delay drug diffusion, and even urge poorly water-soluble drugs to reprecipitate into larger aggregates [42]. Regardless, our findings indicate sustained release of paclitaxel from Abraxane and self-made nanoparticles compared to that from Taxol.

The regression release equation of *in vitro* drug release data for Taxol, Abraxane, and self-made nanoparticles is shown in Table 1. The kinetic patterns of Taxol, Abraxane, and self-made nanoparticles were all fit to the Higuchi equation according to the coefficient, R^2^. The values of n in the Korsmeyer-Peppas equation for Taxol, Abraxane, and self-made nanoparticles were all between 0.45 and 0.89, which indicated that both diffusion and bulk erosion occurred during drug release. This may be ascribed to the low solubility in release media, the diffusion retardation of dialysis, and the interaction between paclitaxel and albumin for Abraxane and self-made nanoparticles.

**Table 1:** Release mechanism of Taxol, Abraxane, and self-made nanoparticles.

Model	Kinetic equation	Fitted parameters	Taxol	Abraxane	self-made nanoparticles
**Zero-order**	Q = kt	R^2^	0.9845	0.9566	0.9564
**First-order**	ln(100-Q) = kt	R^2^	0.9559	0.9822	0.9804
**Higuchi**	Q = kt^1/2^	R^2^	0.9851	0.9876	0.9868
**Korsmeyer-Peppas**	Q = kt^n^	R^2^	0.9793	0.9626	0.9796
		n	0.5865	0.4676	0.4640

#### XRD analysis

**Fig 4:**
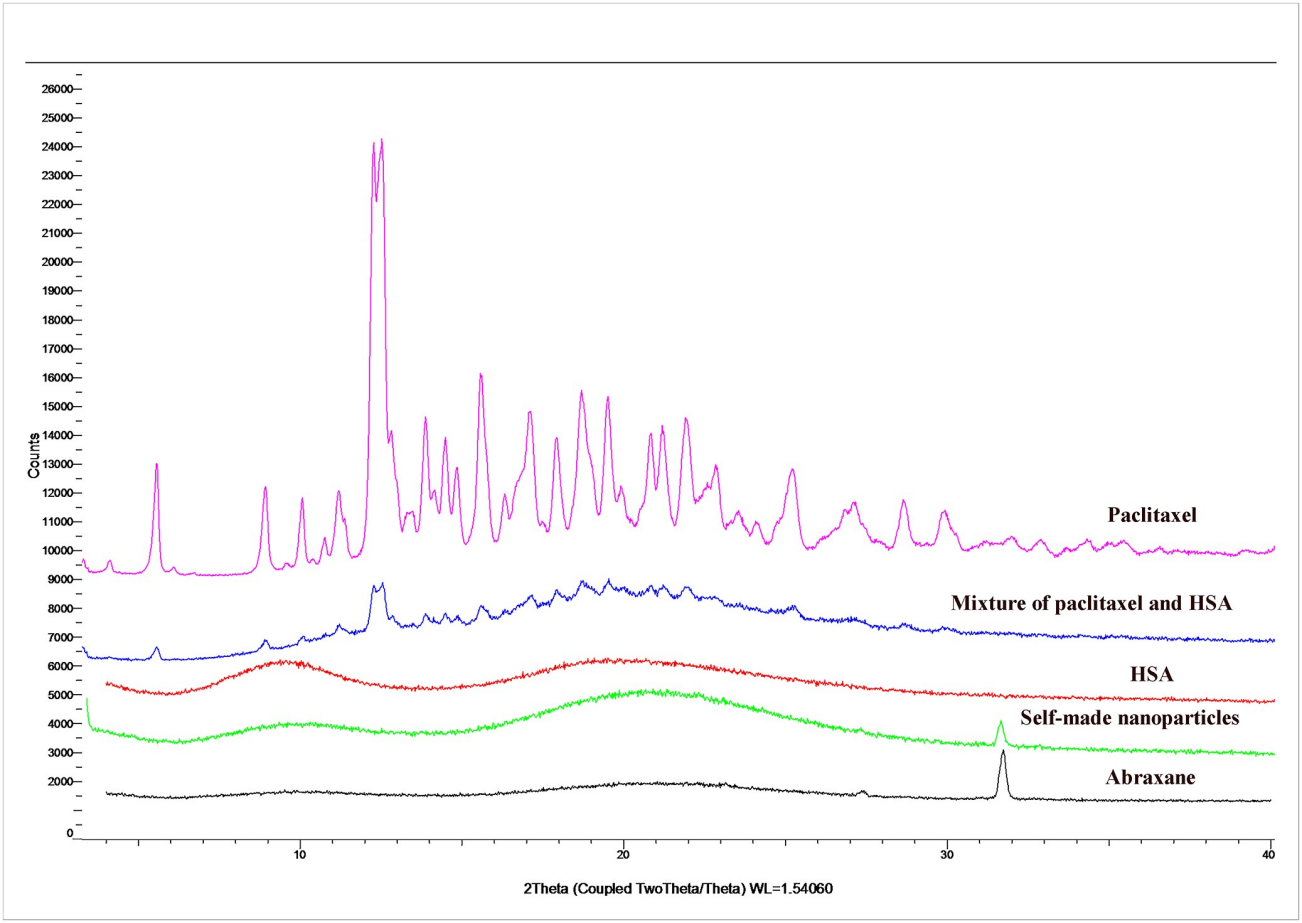
X-ray diffraction patterns of HSA, paclitaxel, physical mixtures of HSA and paclitaxel, Abraxane, and self-made nanoparticles.

XRD is a common method to identify the crystal structures of substances. To evaluate the existing state of paclitaxel in different formulations, HSA, paclitaxel, physical mixtures of HSA and paclitaxel, Abraxane, and self-made nanoparticles were all used for XRD analysis (Fig 4). There were obvious crystal peaks at the same sites in both paclitaxel powders and physical mixtures of paclitaxel and HSA, while these crystal peaks did not emerge in the XRD spectrum of HSA. These results suggest that HSA had no effect on the crystal form of paclitaxel and the physical mixture of paclitaxel and HSA did not affect the existing state of paclitaxel. There were no typical crystal peaks of paclitaxel found in either Abraxane or self-made nanoparticles. Additionally, there was an obvious peak at 32θ in both Abraxane and self-made nanoparticles, whereas this peak did not emerge in paclitaxel powders or the physical mixture of paclitaxel and HSA. These findings suggest that the existing state of paclitaxel in both Abraxane and self-made nanoparticles was changed and that this might be due to the interaction between paclitaxel and albumin. In addition, the XRD profile of paclitaxel in self-made nanoparticles was the same as that of paclitaxel in Abraxane, which suggests that the existing state of paclitaxel in self-made nanoparticles might be coincident with that of paclitaxel in Abraxane [43].

#### DSC analysis

**Fig 5:**
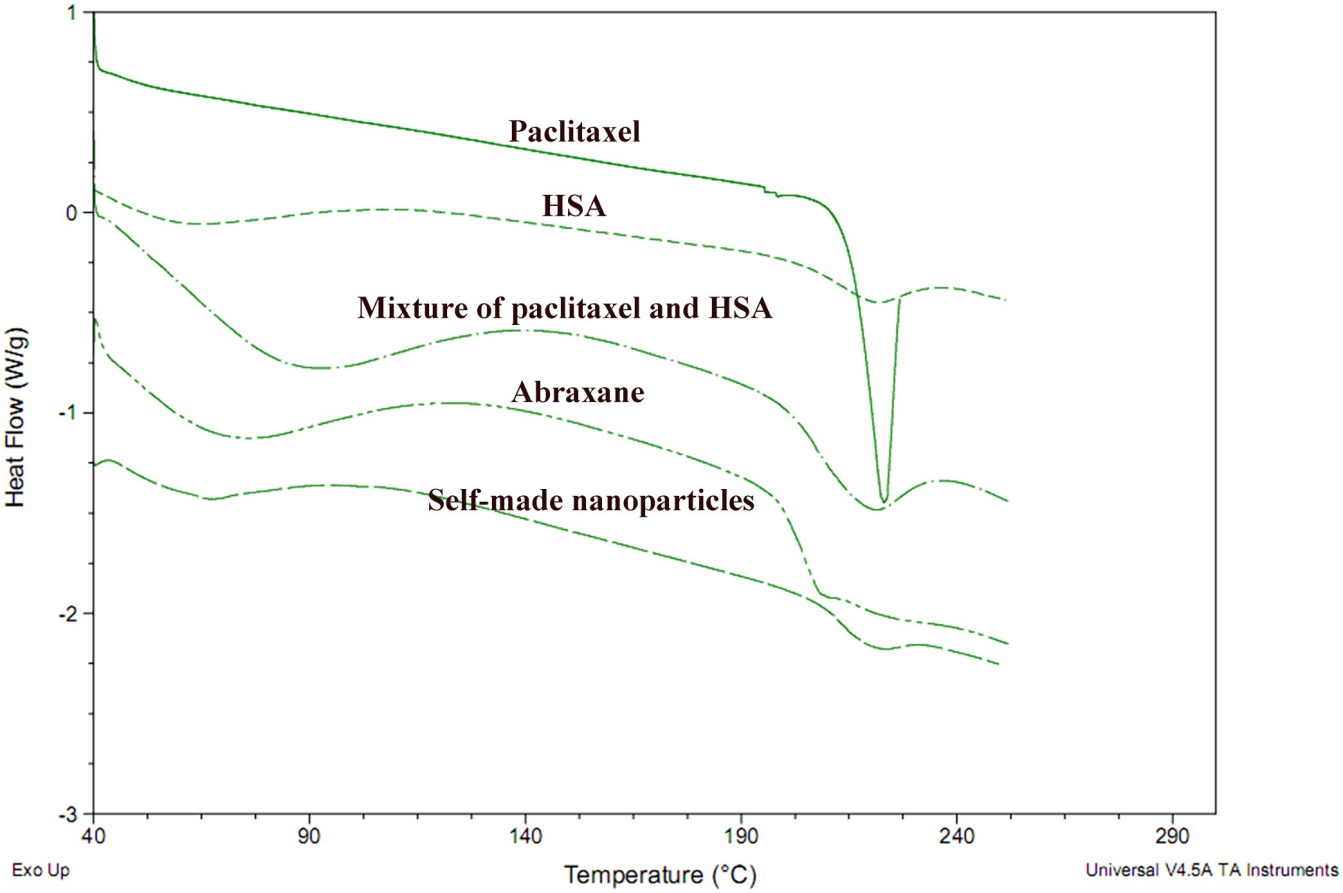
DSC thermograms of paclitaxel, HSA, physical mixtures of paclitaxel and HSA, Abraxane, and self-made nanoparticles.

To further confirm if paclitaxel in self-made nanoparticles existed in the same state as that in Abraxane and other formulations, DSC analysis was performed for paclitaxel powders, HSA powders, physical mixtures of paclitaxel and HSA, Abraxane, and self-made nanoparticles (Fig 5). A melting endothermic peak at approximately 225°C existed in the DSC curve of paclitaxel powders while this melting peak was absent in the curves of Abraxane and self-made nanoparticles, which demonstrated that paclitaxel in both Abraxane and self-made nanoparticles might exist in an amorphous form [44, 45]. Hence, there was no evident difference in the thermodynamic properties of Abraxane and self-made nanoparticles.

#### CD analysis

**Fig 6:**
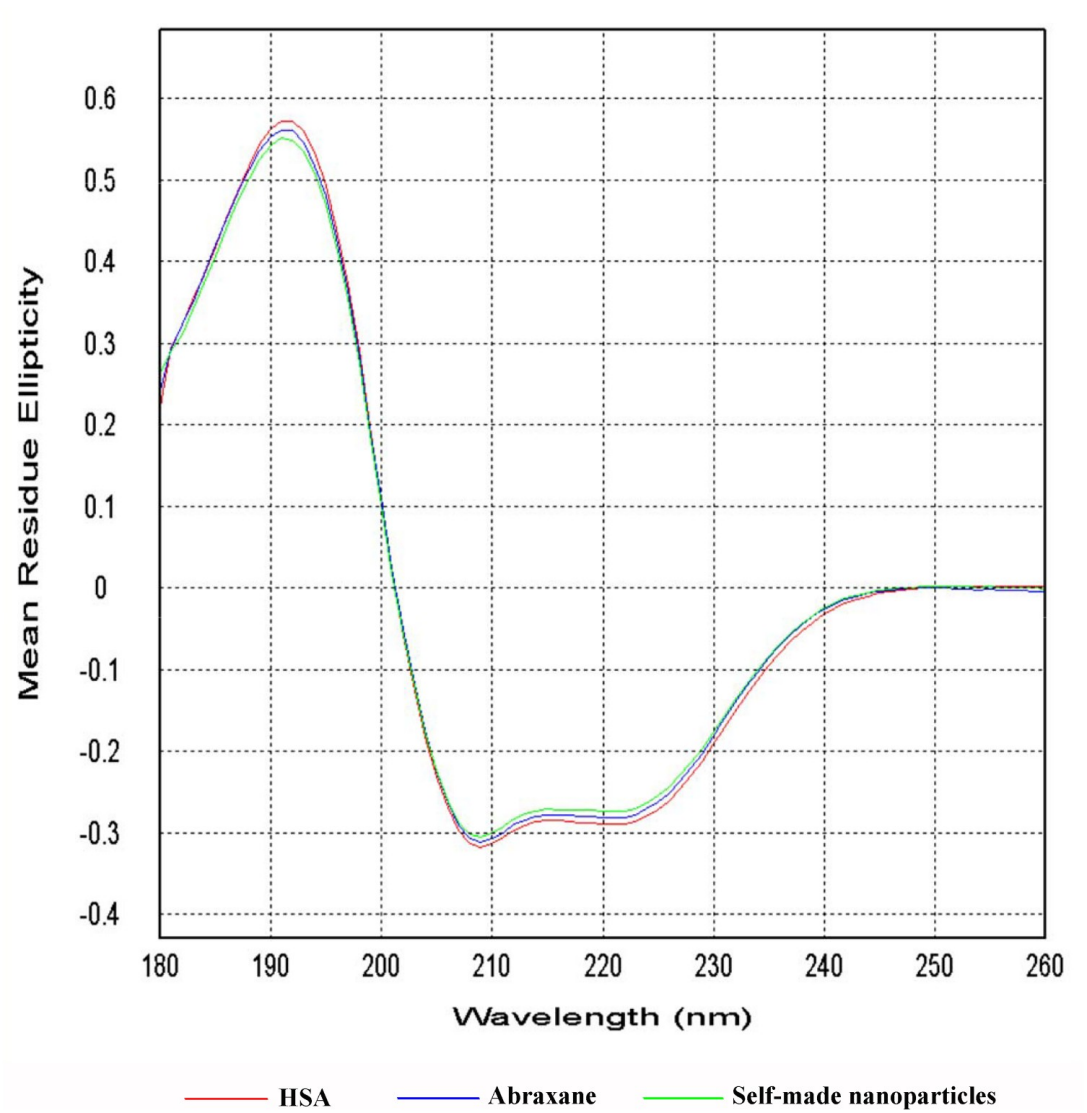
Far-UV CD spectra from 180nm to 260nm for blank HSA and HSA in Abraxane and self-made nanoparticles.

CD is a sensitive technique to monitor conformational changes in proteins. Therefore, far-UV CD spectroscopy was used here in order to evaluate the conformational changes of HSA in Abraxane and self-made nanoparticles. The CD spectra for blank HSA and HSA in Abraxane and self-made nanoparticles are shown in Fig 6, in which the results were expressed as mean residue ellipticity (MRE) in deg cm^2^ dmol^−1^. The CD spectra of HSA in different formulations all exhibited one positive band at 192nm and two negative bands at 208nm and 220nm, and HSA in different formulations did not show a significant shift in the peaks or obvious changes in the intensity of the peaks. All of these findings indicate that the secondary structures of HSA at the surface of Abraxane and self-made nanoparticles were uniform with that of blank HSA [44].

The composition and assignments of the secondary structural elements of HSA were obtained from the CD spectrum between 180nm and 260nm by CDPro software (Table 2). The data revealed that the proportions of helix, antiparallel, parallel, beta-turn, and random coiling in HSA secondary structures were nearly the same for blank HSA, Abraxane and self-made nanoparticles. Therefore, the secondary structures of HSA were similar to those of Abraxane and self-made nanoparticles, and the spatial structures and putative protein functions were consistent with those of blank HSA [46].

**Table 2:** Secondary structure composition and assignments for HSA obtained from CD spectra.

	Helix	Antiparallel	parallel	Beta-Turn	Rndm. Coil	Total Sum
**HSA**	23.20%	19.40%	5.90%	17.00%	38.90%	104.50%
**Abraxane**	23.20%	19.30%	5.90%	17.00%	38.90%	104.30%
**Self-made nanoparticles**	23.30%	19.20%	5.90%	17.00%	38.80%	104.20%

#### Colloidal stability

**Fig 7:**
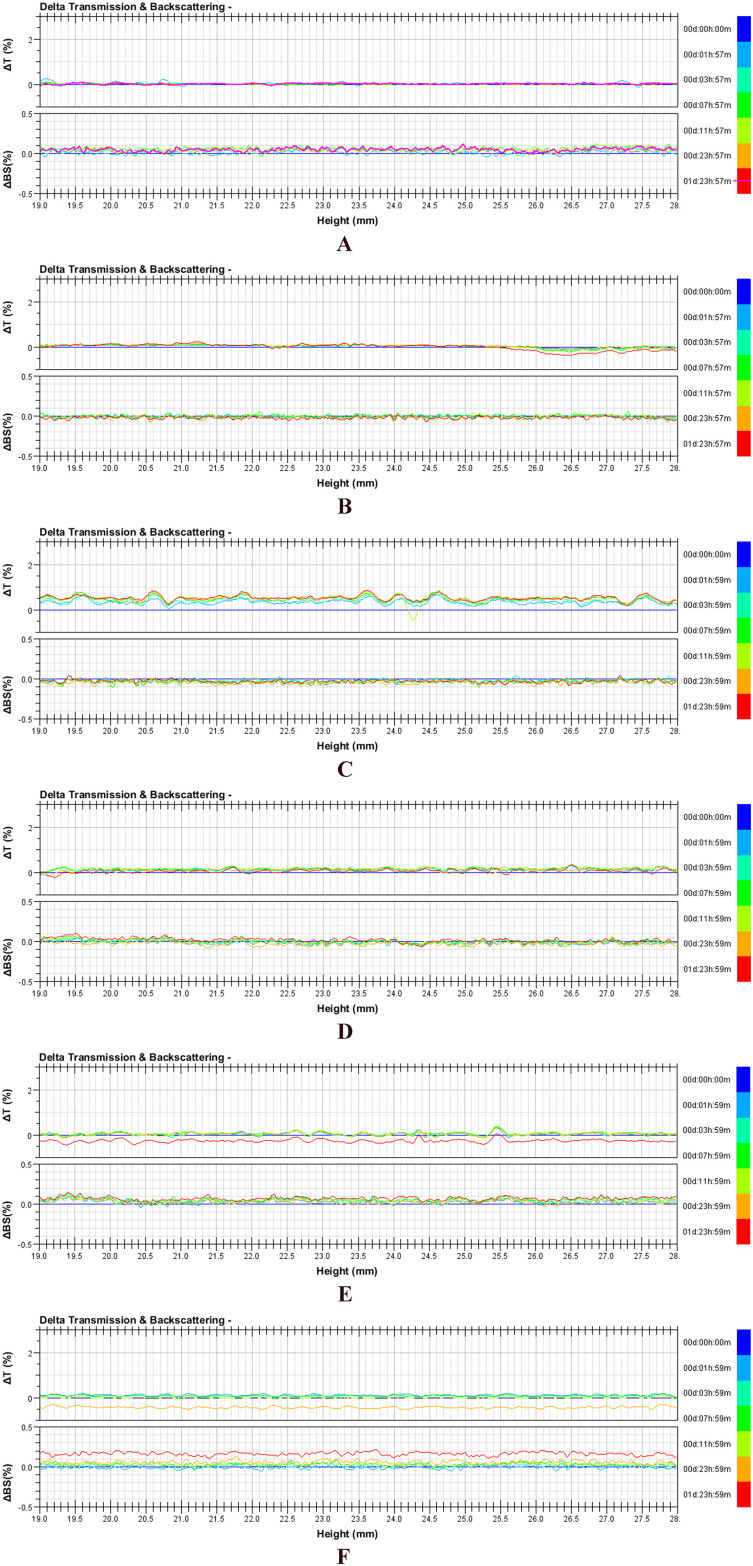
Variation profiles of transmission and backscattering for self-made nanoparticles, Abraxane, and their dilutions at 37°C. (A) Self-made nanoparticles at a paclitaxel concentration of 5mg/mL. (B) Self-made nanoparticles diluted 100-fold with physiological saline. (C) Self-made nanoparticles diluted 100-fold with MEM culture media containing 10% FBS. (D) Abraxane suspension at a paclitaxel concentration of 5mg/mL. (E) Abraxane diluted 100-fold with physiological saline. (F) Abraxane diluted 100-fold with MEM culture media containing 10% FBS.

The colloidal stabilities of self-made nanoparticles, Abraxane, and their dilutions with physiological saline and MEM containing 10% FBS were monitored by Turbiscan Lab Expert, an innovative analytical instrument capable of determining the small changes in colloidal systems. The variation of each formulation in transmission or backscattering profiles during 48h is shown in Fig 7. The results showed that the variations of both transmission and backscattering for all samples were less than 3%, indicating that there was no apparent aggregation, depolymerization or sedimentation that occurred during the culture. Therefore, both self-made nanoparticles and commercially available Abraxane were deemed to be stable *in vitro*.

### Pharmacokinetic study in rats

**Fig 8:**
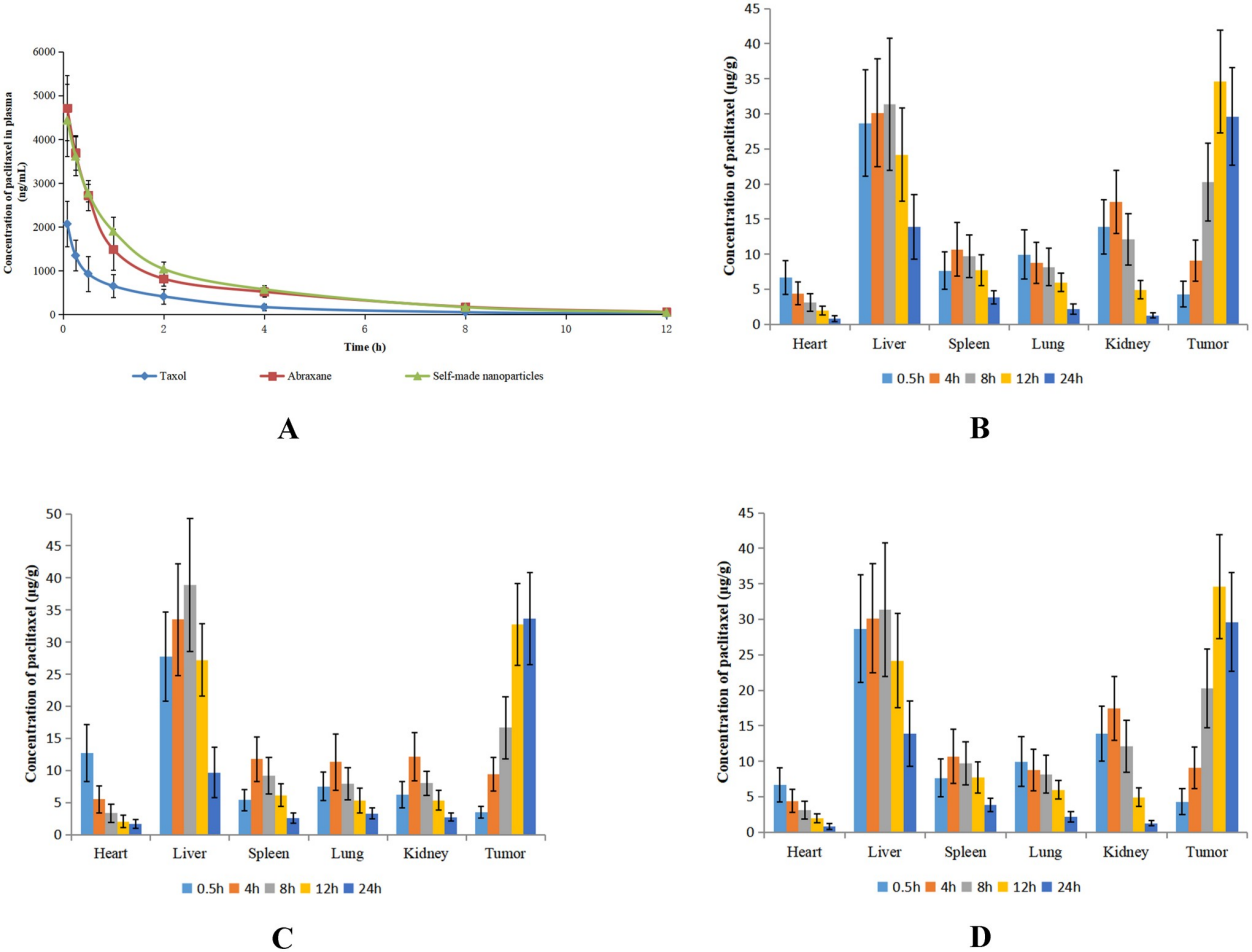
Dynamic and biodistribution of Taxol, Abraxane, and self-made nanoparticles. (A) Plasma concentration-time curves of paclitaxel in rats receiving a single dose of Taxol, Abraxane, or self-made nanoparticles at a paclitaxel dose of 7mg/kg. (B) Biodistribution of paclitaxel in tumor-bearing mice after intravenous administration of Taxol. (C) Biodistribution of paclitaxel in tumor-bearing mice after intravenous administration of Abraxane. (D) Biodistribution of paclitaxel in tumor-bearing mice after intravenous administration of self-made nanoparticles. The data are shown as means ± SD (n = 5).

Paclitaxel concentration-time curves after intravenous administrations of different formulations in rats are shown in Fig 8A. The pharmacokinetic parameters are calculated and shown in Table 3. The concentration-time curves of Abraxane and self-made nanoparticles were mostly overlapping with one another and they were significantly different from that of Taxol, although the concentration of paclitaxel decreased rapidly after administration for all formulations. The maximum plasma concentration (Cmax) and the area under concentration–time curve (AUC) of Taxol were significantly lower than those of Abraxane and self-made nanoparticles (*p*<0.05). This result was perhaps related to the high affinity between albumin and hydrophobic paclitaxel [47]. There was no difference found in CL, Vss, or MRT among the three formulations. Hence, the self-made nanoparticles had similar pharmacokinetic behaviors to those of Abraxane and different pharmacokinetic behaviors from those of Taxol.

**Table 3:** Primary pharmacokinetic parameters of paclitaxel in rats after intravenous administration of Taxol, Abraxane, or self-made nanoparticles at a paclitaxel dose of 7mg/kg (*n* = 5).

Parameters	Taxol	Abraxane	Self-made nanoparticles
**C**_**max**_**(ng/mL)**	2069.3±518.4^b^	4713.3±743.0^a^	4437.8±822.2^a^
**T**_**1/2**_**(h)**	1.97±0.08^b^	2.47±0.13^a^	2.49±0.16 ^a^
**AUC**_**0-inf**_**(μg·h/mL)**	2.87±1.01^b^	7.51±1.46 ^a^	8.23±1.06^a^
**CL(L/h)**	0.67±0.26	0.24±0.05	0.22±0.03
**Vss(L)**	1.57±0.76	0.66±0.13	0.57±0.08
**MRT(h)**	2.30±0.26	2.75±0.05	2.66±0.12

^a^ *p*<0.05 compared with Taxol;

^b^ *p*<0.05 compared with Abraxane.

### Biodistribution study in mice

The distribution of paclitaxel in tumors and primary organs of mice at different time were investigated after intravenous administrations of Taxol, Abraxane, and self-made nanoparticles and the results are shown in Fig 8. At the 0.5-h time point after administration of Taxol (Fig 8B), highly perfused systemic tissues–such as the liver, lung, kidney, spleen, and heart–showed relatively high drug concentrations. The concentration of paclitaxel in these organs reached a maximum at 4h and then decreased slowly. In contrast to the tendency of paclitaxel in primary organs, the concentration of paclitaxel in tumors increased with time until 12h after administration. The highest concentration of paclitaxel among all organs and tissues was observed in the liver, as this drug concentration was significantly higher than those in other organs or tissues. For mice treated with Abraxane (Fig 8C), paclitaxel was distributed into different organs soon after intravenous administration and the drug concentration reach a maximum before 4h, except in the liver and in tumors. The paclitaxel concentrations in liver and tumors were much higher than those in other organs and the paclitaxel concentrations in these two tissues were almost equivalent with one another. The maximum paclitaxel concentration in tumors of mice given Abraxane was approximately 2–3 fold higher than that in tumors of mice given Taxol. Therefore, the distribution of paclitaxel in tumors was significantly improved for Abraxane compared with that of Taxol. The distribution of paclitaxel in mice given self-made nanoparticles (Fig 8D) was similar to that in mice administered Abraxane and there was no difference between paclitaxel concentrations in tumors of mice given Abraxane and self-made nanoparticles. Hence, both Abraxane and self-made nanoparticles markedly improved the distribution of paclitaxel in tumors.

Evident differences in pharmacokinetic between Abraxane and Taxol have been reported in previous studies [48–50]. In Taxol formulation, CrEL has been added to enhance the solubility of paclitaxel. However, CrEL alters the disposition of paclitaxel by entrapping paclitaxel into formed micelles, impeding drug delivery to tissue and consequently reducing drug exposure to tumors [49, 50]. By comparison, the faster and more extensive distribution of paclitaxel into tissue compartments and higher accumulation in tumors have been observed for Abraxane. This may be due to nab paclitaxel utilizing endogenous transport pathways of albumin to achieve enhanced drug distribution in tumor tissues. There are two reasons for the improved tumor distribution caused by albumin. One reason is that albumin can be transported across the endothelial barrier of blood vessels through binding to gp60 albumin receptor and activating caveolae-mediated endothelial transcytosis [51–53]. The other reason is that tumor cells use albumin as a major energy and nitrogen source through endocytosis and lysosomal degradation, which results in a high accumulation of albumin in tumors [54, 55].

### Anti-tumor efficacy in mice

**Fig 9:**
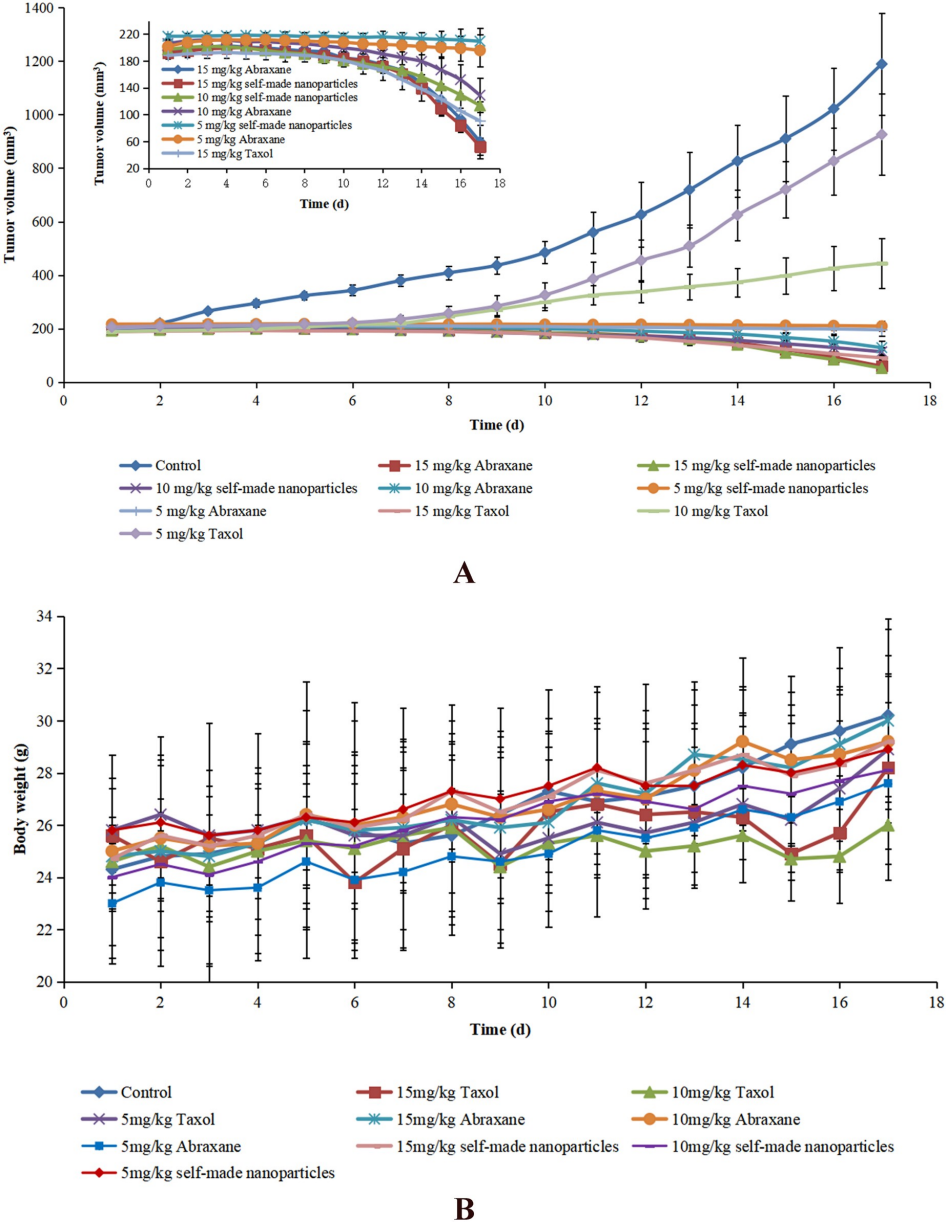
Anti-tumor effects of different paclitaxel formulations after intravenous administration to H22 tumor-bearing mice. (A) Anti-tumor efficacy in H22 tumor-bearing mice after treatment with Taxol, Abraxane, and self-made nanoparticles at different doses (5mg/kg, 10mg/kg and 15mg/kg). (B) Body weight changes of H22 tumor-bearing mice after treatment with Taxol, Abraxane, and self-made nanoparticles at different doses (5mg/kg, 10mg/kg and 15mg/kg). Data are presented as means ± SD (n = 5).

The anti-tumor activities of Taxol, Abraxane, and self-made nanoparticles were evaluated, and the results are shown in Fig 9A and Table 4. The volume of tumors in mice receiving physiological saline (as controls) increased rapidly during the whole period while the growth of tumors in mice administered Taxol, Abraxane, and self-made nanoparticles was significantly inhibited. The anti-tumor activity was evidently dose-dependent in spite of paclitaxel formulation because the anti-tumor efficacy increased with the increased dose of administration. The anti-tumor activities of both Abraxane and self-made nanoparticles were stronger than those of Taxol at the same dose, and the inhibitory efficacies of both Abraxane and self-made nanoparticles at 5mg/kg were nearly equal to that of Taxol at a dose of 10mg/kg. In addition, the highest inhibitory effect above 95% was observed in mice administered Abraxane or self-made nanoparticles at the dose of 15mg/kg. All of these results indicate that the anti-tumor activity of paclitaxel was significantly improved through the introduction of albumin nanoparticles, which is likely related to enhanced drug distribution in tumor tissues caused by albumin. There was no significant difference in the anti-tumor activities of Abraxane and self-made nanoparticles at the same dose. This was considered reasonable since there was no difference found in the appearance, particle size, secondary structure of albumin, release of paclitaxel, colloidal stability, pharmaceutical behavior, or biodistribution between Abraxane and self-made nanoparticles.

**Table 4:** Volume, mass and inhibition rate of tumor (n = 5).

Group	Mass (g)	Inhibition Rate (%)
**Physiological saline (CG)**	2.15±0.40	-
**5mg/kg Taxol**	1.59±0.40	26.40
**10mg/kg Taxol**	0.78±0.14^a^^,^^b^	63.93
**15mg/kg Taxol**	0.17±0.02 ^a^^,^^b^^,^^c^	92.34
**5mg/kg Abraxane**^**®**^	0.71±0.19 ^a^^,^^b^	67.26
**10mg/kg Abraxane**^**®**^	0.24±0.03 ^a^^,^^b^^,^^c^^,^^e^	89.01
**15mg/kg Abraxane**^**®**^	0.08±0.02 ^a^^,^^b^^,^^c^^,^^d^^,^^e^^,^^f^	96.41
**5mg/kg self-made nanoparticles**	0.53±0.08 ^a^^,^^b^	75.62
**10mg/kg self-made nanoparticles**	0.19±0.02 ^a^^,^^b^^,^^c^^.^^d^^,^^e^	91.36
**15mg/kg self-made nanoparticles**	0.05±0.01 ^a^^,^^b^^,^^c^^,^^d^^,^^e^^,^^f^	97.58

^a^*p*<0.05 compared with physiological saline;

^b^*p*<0.05 compared with 5mg/kg Taxol;

^c^*p*<0.05 compared with 10mg/kg Taxol;

^d^*p*<0.05 compared with 15mg/kg Taxol;

^e^*p*<0.05 compared with 5mg/kg Abraxane^®^;

^f^*p*<0.05 compared with 10mg/kg Abraxane^®^.

The body weight and health status of each mouse were also monitored during the whole treatment period to evaluate the toxicity of different formulations. As shown in Fig 9B, a transitory decrease of body weight was observed in mice administered drugs in all formulations while this phenomenon was not found in mice administered physiological saline, which indicated that this weight loss may have been caused by the administration of paclitaxel. Mice treated with Taxol showed significant weight loss and appeared unhealthy, especially for those receiving Taxol at a high dose, which indicated that the most serious side effects occurred in mice administered Taxol. A slight loss of body weight was shown in mice that received Abraxane and self-made nanoparticles with different dosages and there were no noteworthy changes in health for these mice. Therefore, Abraxane and self-made nanoparticles were found to be safer than Taxol [22, 56].

**Fig 10:**
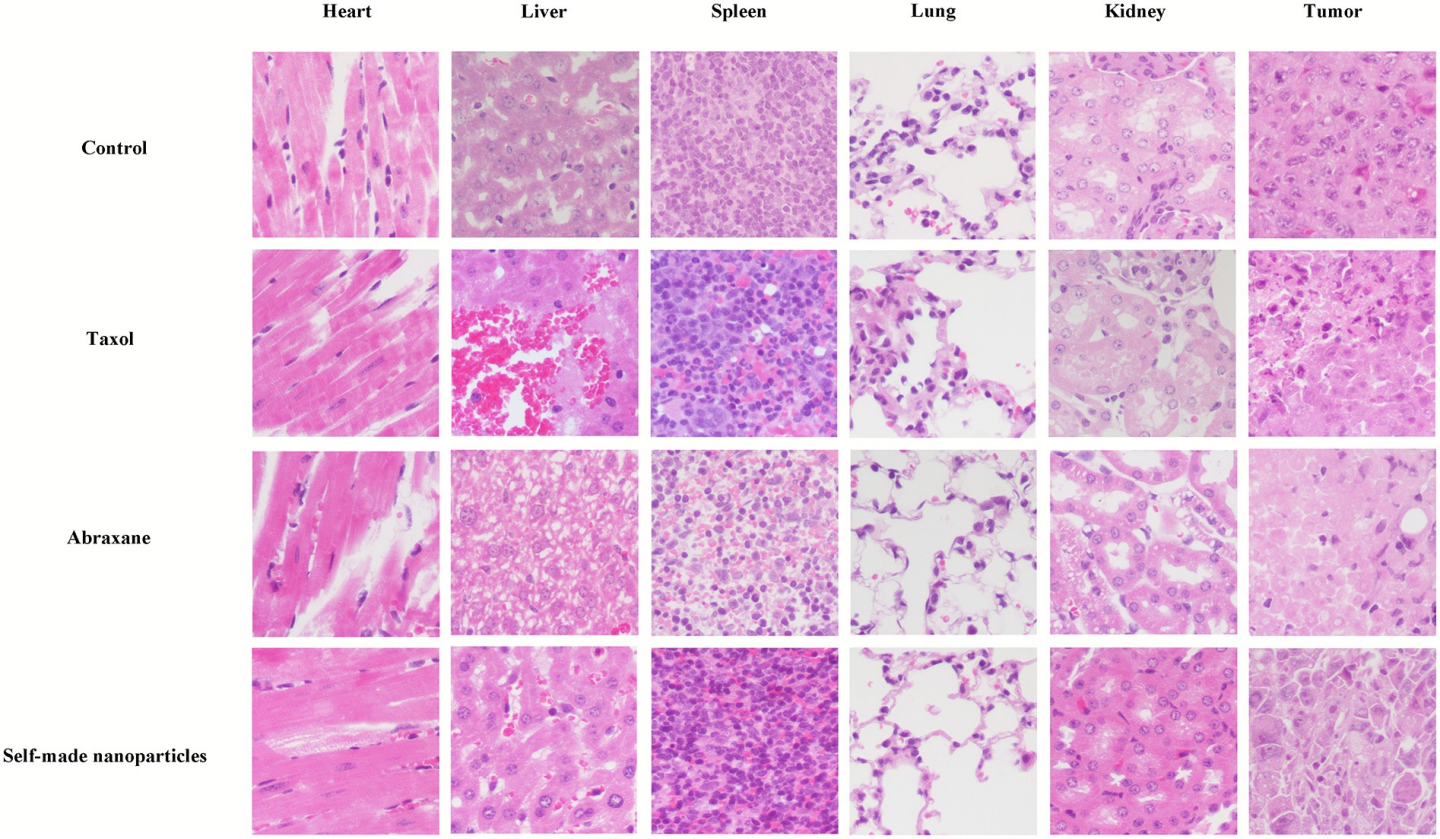
Photographs of primary organs and tumor tissues stained with H&E from mice treated with physiological saline, Taxol, Abraxane, and self-made nanoparticles at the dose of 10mg/kg (20×).

Histological transmutations of deprived organs and tumor tissues stained with H&E are displayed in Fig 10. Tumor cells appeared integral, karyokinesis was obvious, and primary organs–including the heart, liver, spleen, lung and kidney–appeared normal in the control group. Significant cellular necrosis in tumors was observed in mice treated with paclitaxel at different formulations, and the necrotic regions in tumors were approximately 40%, 90%, and 90%, for mice treated with Taxol, Abraxane, and self-made nanoparticles, respectively. These results reflected that the treatment of paclitaxel in all formulations could significantly inhibit tumors. However, the anti-tumor efficacies of formulations constructed with albumin (Abraxane and self-made nanoparticles) were stronger than that of the formulation with high Cremophor EL (Taxol), and there was no difference in tumor inhibition between Abraxane and self-made nanoparticles. This was consistent with our results of tumor growth. The hepatic cells looked normal, and there was no cellular necrosis or inflammatory cell infiltration found in the livers of the control group. However, massive hemorrhage, necrosis, and degenerative changes (e.g. loose cytoplasm) were observed in liver tissues of mice given Taxol. Degenerative changes were also found in livers of mice administered Abraxane and self-made nanoparticles while cellular necrosis or inflammatory cell infiltration were not observed. There was no significant change found in other tissues (heart, spleen, lung, and kidney) of mice administered Taxol, Abraxane, or self-made nanoparticles. Therefore, these findings indicated that Taxol exhibited more serious side effects in the liver than the other treatments, whereas there was no difference in the side effects between Abraxane and self-made nanoparticles.

## Conclusions

We developed and validated a novel preparative method for nab paclitaxel. This method was based on solubilization of paclitaxel in liquid PEG and interaction between paclitaxel and albumin in PEG and aqueous solution. Therefore, the principle and preparative procedures of our novel method are completely different from those of previously reported methods, which is also proved by the authorization of patent from both China and Japan. These self-made nab paclitaxel acquired 50% higher drug loading than Abraxane and undetected ethanol residue, and the high consistency between behaviors of self-made nanoparticles and Abraxane was also observed both *in vitro* and *in vivo*. In a word, our novel preparative method for nab paclitaxel can significantly improve drug loading, obviously decrease product cost, and is considered to have potent practical value.
